# Effect of the Ratio of Protein to Water on the Weak Gel Nonlinear Viscoelastic Behavior of Fish Myofibrillar Protein Paste from Alaska Pollock

**DOI:** 10.3390/gels10110737

**Published:** 2024-11-13

**Authors:** Timilehin Martins Oyinloye, Won Byong Yoon

**Affiliations:** 1 Department of Food Science and Biotechnology, College of Agriculture and Life Sciences, Kangwon National University, Chuncheon 24341, Republic of Korea; oyinloyetm@kangwon.ac.kr; 2 Elder-Friendly Research Center, Agriculture and Life Science Research Institute, Kangwon National University, Chuncheon 24341, Republic of Korea

**Keywords:** fish myofibrillar protein, weak gel properties, rheology, SAOS, LAOS, shear thinning, shear thickening, protein–protein interactions

## Abstract

The linear and nonlinear rheological behaviors of fish myofibrillar protein (FMP) paste with 75%, 82%, and 90% moisture content were evaluated using small-amplitude oscillatory shear (SAOS) and large-amplitude oscillatory shear (LAOS) tests. SAOS revealed pastes with 75% and 82% moisture exhibited solid-like behavior, characterized by higher storage modulus (G′) than loss modulus (G″), indicative of weak gel properties with a strong protein interaction. In contrast, the 90% moisture content showed more viscous behavior due to weakened protein–protein entanglements. The frequency exponent (n′ and n″) from the power law equation varied slightly (0.24 to 0.36), indicating limited sensitivity to changes in deformation rate during SAOS. LAOS tests revealed significant structural changes, with Lissajous–Bowditch curves revealing early nonlinearities at 10% strain for 90% moisture content. Decomposed Chebyshev coefficients $(e_{3}/e_{1},\;v_{3}/v_{1},\;S,\;\mathrm{and}\;T)$ indicated strain stiffening at lower strains for the 75% and 82% moisture pastes (i.e., < 50% strain for 75% and < 10% strain for 82%), transitioning to strain thinning at higher strains. Additionally, numerical model confirmed the predictability of the 3D printing process from the nonlinear rheological data, confirmed the suitability of the 75% and 82% moisture pastes for applications requiring structural integrity. These insights are essential for optimizing processing conditions in industrial applications. The findings suggest that the 75% and 82% moisture pastes are suitable for applications requiring structural integrity, while the 90% moisture paste is ideal for flow-based processes. These insights are essential for optimizing processing conditions in industrial applications.

## 1. Introduction

Dynamic oscillatory shear flow measurements are key to examining the elastic and viscous properties of viscoelastic materials like biopolymers, suspensions, emulsions, gels, and food materials [1,2]. This test uses various modes, with small amplitude oscillatory shear (SAOS) and large amplitude oscillatory shear (LAOS) being the most prevalent [3]. While SAOS effectively analyzes materials in their linear viscoelastic region (LVR) [4,5], it fails to capture the complex rheological behavior of food materials under the large, rapid deformations encountered in processing and preparation [6]. In protein pastes with gel-like structures, pre-existing protein networks show unique viscoelastic characteristics under stress, influencing stability in industrial processes. Capturing these nonlinear responses is essential for materials transitioning between pastes and weak gels, requiring measurements beyond traditional linear approaches [6].

Ewoldt et al. [7] introduced a methodology for analyzing rheological behavior in gels and structured food systems using Fourier transforms and stress signals in Chebyshev polynomials. This approach enhances understanding of nonlinear viscoelastic responses in gel-like systems, such as strain stiffening/softening and shear thickening/thinning, which are critical for assessing food gel stability during processing, beyond SAOS capabilities. Large deformations and nonlinear rheological parameters (stiffening ratio (S), thickening ratio (T), dynamic modulus at maximum shear rate (${G^{\prime }}_{M}$), and at maximum strain (${G^{\prime }}_{L})$) reveal how materials respond to significant deformations in processes like mixing, extrusion, and coating [8,9]. For example, high strain stiffening (positive S) resists deformation, maintaining structure in mixing, while positive T values indicate shear thickening, affecting flow in extrusion [10]. LAOS measurements have become vital for studying nonlinear viscoelastic behaviors in complex food materials like gels, emulsions, foams, and suspensions [11,12,13,14].

Fish myofibrillar protein (FMP) concentrate, known as “*Surimi*”, is a salt-soluble protein with unique gelling properties ideal for seafood products. While FMP can be derived from various fish species, Alaska pollock (*Theragra chalcogramma*) offers high processing yield, strong gel-forming ability, mild flavor, and low fat [15,16]. FMP in its initial paste state, before gelation forms a loosely entangled protein network, giving it weak gel-like properties that impart viscoelastic behavior beyond classical fluid mechanics. This structure provides elasticity and resilience under stress, essential for manufacturing processes like mixing and extrusion in seafood production. While the fully gelled FMP has been studied for rupture strength and viscoelasticity, the paste state with its weak gel-like properties remains underexplored [17,18,19]. In co-extrusion, surimi often requires high moisture (82–90%) for optimal mechanical and sensory qualities [16]. Understanding the linear and nonlinear viscoelastic responses of high-moisture, weak-gel-like FMP is thus critical for its industrial applications [19,20].

Numerical modeling has become invaluable in predicting the outcomes of complex industrial processes, such as extrusion, mixing, molding and 3D printing [19]. These models rely on accurate material data to simulate process dynamics effectively, and nonlinear rheological properties play a critical role in these predictions. Unlike linear viscoelastic properties, which offer limited insight into material stability under small deformations, nonlinear properties provide a more accurate representation of material behavior under high strains [8,9]. Studies by Hyvärinen et al. [21] and Melito et al. [11] emphasize that incorporating nonlinear data in modeling enhances process control, product consistency, and quality, particularly in high-strain applications. For example, LAOS analysis has been used to optimize dough leavening time, mixing, and shaping in the baking industry, improving product consistency and quality [9]. Thus, nonlinear viscoelastic properties are crucial for understanding the behavior of FMP paste in industrial applications. Without incorporating nonlinear rheology, numerical models based solely on SAOS data fall short in simulating complex flow and structural responses in high-strain conditions, underscoring the need for both linear and nonlinear data to fully capture material behavior in industrial processes.

Therefore, the objectives of this study were as follows: (1) to characterize the linear and nonlinear viscoelastic responses of FMP paste having weak gel properties with varying moisture content; (2) to examine how moisture content influences FMP paste’s viscoelastic properties, especially its protein network structure; (3) to apply the Chebyshev stress decomposition method in the interpretation of elastic and viscous nonlinearity of FMP paste, including the identification of strain stiffening and thinning behavior; (4) to evaluate the industrial relevance of these rheological properties for optimizing processing parameters in mixing, extrusion, and molding; and (5) to validate the importance of FMP paste’s nonlinear viscoelastic properties in industrial applications like 3D printing through numerical modeling.

## 2. Results and Discussion

### 2.1. Linear Rheological Behavior of FMP Paste

#### 2.1.1. Strain Sweep Analysis and the Influence of Moisture Content on the Linear Viscoelastic Region (LVR) of FMP Paste

**Figure 1:**
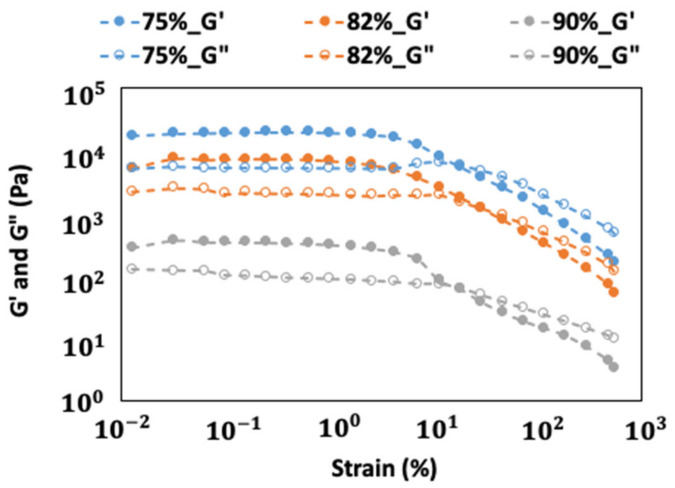
Strain sweep analysis for FMP paste with different moisture contents, obtained at frequency 1 Hz.

Strain sweep analysis was used to determine the LVR of FMP paste at different moisture contents (Figure 1). The result demonstrated that at lower moisture content (75%) and lower strain, the storage modulus (G′) was significantly higher than the loss modulus (G″), indicating that FMP paste behaves as a yield-stress liquid with gel-like characteristics, where elastic behavior predominates over viscous behavior within the yield-stress domain. This behavior reflects the entanglement of myofibrillar proteins, particularly myosin and actin, which form the structural backbone of the paste. Myosin’s long rod-like tail (light meromyosin region) and globular head enable entanglement even without heat [22,23]. These weak gel properties arise from van der Waals forces, hydrogen bonding, and electrostatic interactions, resulting in a denser matrix that resists deformation, as seen in the broader LVR range (approximately 0.01% to 4% strain) [20]. Limited water availability enhances protein hydration and entanglement, creating a mechanically stable structure capable of withstanding higher strains. These findings align with Yoon et al. [16], who noted that reduced water availability promotes protein–protein interactions and network formation. Kanmani and Rhim [24] also discussed how less free water increases protein proximity, enhancing network stability. Thus, the paste’s weak gel properties allow it to endure higher strains before yielding, as reflected in the extended LVR (Figure 1).

As the moisture content increases to 82%, the paste becomes less rigid, as shown by the reduced G′ values and a shortened LVR (0.01 to 2%). Increased water promotes protein hydration, disrupting protein entanglements and enhancing paste flexibility. Water also acts as a plasticizer, increasing protein mobility and loosening the entangled structure, reducing the paste’s resistance to strain [25]. This aligns with the observation of Kuang et al. [26], who found that higher moisture in pea protein isolate weakens network cohesion due to diminished protein–protein interactions, leading to more fluid-like behavior.

At 90% moisture content, the paste exhibited the lowest G′ values and shortest LVR (0.01 to 1.5% strain), dominated by protein–water interactions. Proteins are less entangled, forming a sparse, fluid-like network that yields more easily under strain. Ghanbarpour et al. [27] also noted that high hydration reduces network density and stability, reflecting the impact of moisture content on FMP paste’s viscoelastic properties. Consequently, a strain of 0.1% was selected for the frequency sweep analysis, as this value lies comfortably within the LVR for all moisture content ranges, allowing for accurate characterization of the linear rheological behavior of FMP paste without inducing nonlinear behaviors that could compromise the results. This approach facilitates a reliable assessment of the frequency-dependent rheological behavior of the paste, providing critical insights into how moisture content influences its weak gel viscoelastic properties.

#### 2.1.2. Effect of Moisture Content on the Frequency-Dependent Viscoelastic Behavior of FMP Paste

**Figure 2:**
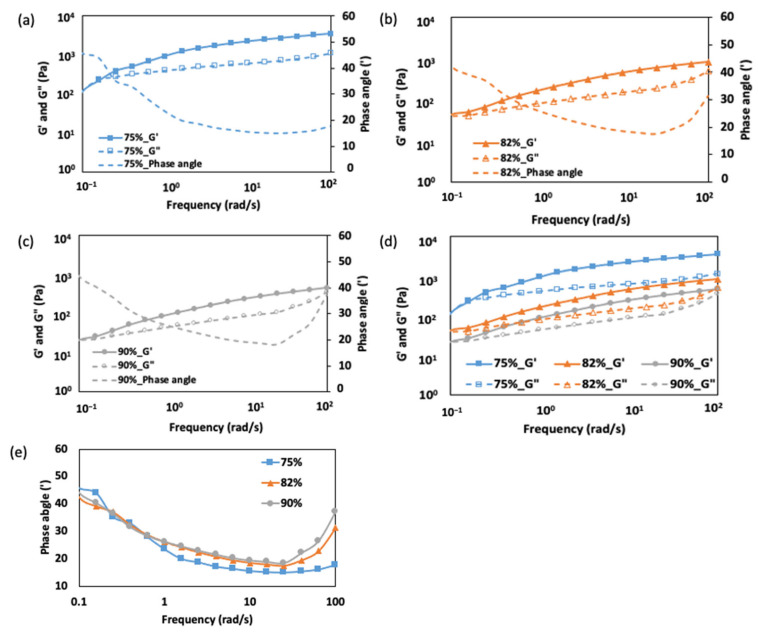
Rheological properties of the FMP paste according to moisture content: (**a**) 75%, (**b**) 82%, (**c**) 90% (G′, G″, phase angle), (**d**) combined G′ and G″ for all moisture contents, and (**e**) combined phase angle for all moisture contents.

The frequency sweep analysis of FMP paste at 0.1% strain (Figure 2a–d), reveals how moisture content influences its viscoelastic behavior. Increasing moisture content generally leads to a transition from elastic-dominated behavior to more fluid-like characteristics. This shift is due to the balance between protein–protein interactions (entanglements) and protein–water interactions, which significantly impacts the structural integrity of the protein network and its resistance to deformation. At 75% moisture content, the paste exhibits a predominantly elastic behavior, indicated by lower phase angles (Figure 2e). This is due to a dense network of protein entanglements, particularly from myosin flexible tails [28]. Limited water enhances protein–protein interactions through hydrogen bonds and hydrophobic attractions, resulting in paste with gel-like characteristics having higher G′ and minimal energy dissipation [29].

As the moisture content increases to 82% and 90% (Figure 2b,c), the paste shifts toward fluid-like behavior as water disrupts protein entanglement and increases molecular mobility [28]. This leads to a decrease in G′ and an increase in G″, indicating greater susceptibility to deformation and flow. Lower phase angles at these moisture levels confirm the transition to viscous-dominated behavior, especially at low frequencies (<0.3 rad/s), where the material can rearrange and flow (Figure 2e).

At low frequencies (<1 rad/s), the material’s response time allows for relaxation, with the viscous component dominating at higher moisture levels (82% and 90%). This is reflected in the higher phase angles observed at low frequencies, as the loosely bound network of hydrated proteins exhibits more fluid-like behavior [16]. Conversely, at high frequencies (40–100 rad/s), rapid deformation enhances the elastic response, especially at 75% moisture, where the entanglement network resists deformation. For the 82% and 90% moisture contents, higher phase angles suggest a weaker elastic response due to increased hydration and reduced entanglement, resulting in greater energy dissipation and more viscous flow. This is consistent with findings in high-moisture protein systems, like soy protein, where increased water content disrupts the protein matrix and enhances the fluidity [30,31].

Overall, the frequency sweep analysis and phase angle results highlight the critical role of moisture in determining FMP paste’s viscoelastic properties. Lower moisture levels yield paste with weak gel-like properties below the yield stress, while higher moisture contents lead to fluid-like characteristics dominated by protein–water interactions. These findings align with other protein-based pastes, where moisture acts as a plasticizer, reducing network density and increasing fluidity [32].

#### 2.1.3. Damping Factors and Frequency Dependence of FMP Paste at Varying Moisture Contents

**Figure 3:**
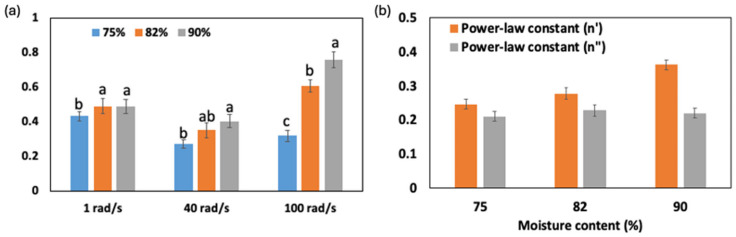
Damping factor (G″/G′) for FMP paste at different frequencies (1 rad/s, 40 rad/s, and 100 rad/s) and moisture contents (75%, 82%, and 90%) (**a**), and power law constants (n′ and n″) for FMP paste at different moisture contents (**b**). Different lowercase letters indicate significant differences within the same measurement factor (*p* < 0.05).

Figure 3a shows the damping factors (G″/G′) for FMP paste at frequencies of 1, 40, and 100 rad/s. The damping factor is crucial, as it indicates the ratio of viscous to elastic response; a higher value denotes more viscous behavior. Since the phase angle (tan δ) is not a monotonous function of frequency (as shown in Figure 2e), analyzing damping factors at different frequencies provides insight into the weak gel-like properties of the paste across various conditions: within the LVR (1 rad/s), at the onset of elasticity reduction (40 rad/s), and at higher frequencies (100 rad/s). This comparison can reveal how moisture content influences transitions from solid-like to fluid-like behavior [33].

At 1 rad/s, damping factors range from 0.43 (75% moisture) to 0.49 (82% and 90%), indicating that higher moisture enhances protein–water interactions, thus increasing molecular mobility and viscosity. At 40 rad/s, the damping factor for the 75% moisture paste remains at 0.27, suggesting gel-like behavior, while higher moisture levels (82% and 90%) show increased damping factors (0.35 and 0.40), signaling a shift to fluid-like behavior as water disrupts protein–protein entanglements. This aligns with findings by Li et al. [13], who observed that lower moisture enhances cohesion in protein networks. At 100 rad/s, the damping factor rises to 0.31 for 75%, 0.61 for 82%, and 0.76 for 90%, reflecting increased fluidity with moisture, where the higher water content reduces elasticity and facilitates flow, consistent with findings in surimi-based products [34].

The power law model in Figure 3b provides additional insights into frequency dependence, particularly for the storage modulus (G′). At 75% moisture, the lower n′ (0.247) suggests elasticity is minimally frequency-dependent, reflecting gel-like behavior with strong protein–protein interactions. A slight viscous increase (n″ = 0.211) reflects low molecular mobility, as seen in other protein systems where lower moisture enhances network strength [35]. Although this model describes the behavior of G′ well, the loss modulus (G″) may exhibit more complex, non-linear responses to frequency, especially at higher moisture contents. This is due to the influence of protein–water interactions, which can lead to changes in energy dissipation mechanisms and disrupt the entangled network, causing deviations from a strict power law trend. With 82% moisture, both n′ and n″ increase (to 0.278 and 0.228, respectively), suggesting a more frequency-sensitive elastic modulus due to protein network loosening, with enhanced mobility and fluid-like behavior. This trend is supported by the findings of Yoon et al. [16], who observed a similar effect in surimi-based system, where increased moisture content led to a more fluid-like behavior due to the loosening of the protein network. At 90% moisture, the sharp rise in n′ to 0.362 shows highly frequency-dependent elasticity, as water disrupts the protein matrix, allowing significant rearrangement under shear. However, n″ remains relatively stable at 0.220, indicating less frequency sensitivity in viscous behavior, likely due to water’s lubricating effect. This contrasts with low-moisture pastes, where viscosity plays a minimal role.

In summary, the power-law model shows that increased moisture content makes the elastic response of FMP paste more sensitive to frequency, while the viscous response changes less significantly. This shift weakens the paste’s gel-like structural integrity, allowing greater deformation under oscillatory shear, especially at higher frequencies. Understanding these changes is critical for tailoring the rheological properties of protein paste in industrial applications. In low-frequency processes like slow-speed mixing, where viscous behavior dominates (G″ > G′), FMP paste with higher moisture can flow easily, minimizing elastic recoil. In medium-frequency operations (1–10 rad/s), balancing viscous and elastic behavior aids in efficient processing without compromising structure. At higher frequencies (> 10 rad/s), processes like high-speed molding can utilize increased elasticity (G′ > G″) of FMP paste, especially at lower moisture contents, to maintain shape during extrusion. This behavior, typical of gel-like materials, is less common in food pastes. However, careful frequency selection is essential to avoid elastic recoil that could disrupt product formation.

These findings also have implications for energy efficiency. Lower frequencies minimize energy consumption but may prolong processing times, while higher frequencies can speed up processing but require robust equipment and greater energy input. By fine-tuning parameters based on rheological properties like the LVR and damping factor, manufacturers can optimize conditions that balance material behavior, efficiency, and product quality. Since FMP paste exhibits weak gel properties, studying its non-linear rheological behavior is essential for understanding responses under extreme strain and shear. While the LVR reveals behavior under small deformations, real-world applications often exceed these conditions, where the material’s structure may break down. Non-linear analysis will provide deeper insights into yielding behavior and strain effects, vital for predicting performance during large deformations in extrusion or high-speed mixing. Understanding non-linear rheology will enhance control over processing conditions, ensuring consistency, texture, and structural integrity across various manufacturing conditions. These insights are particularly relevant for food processing, extrusion, and molding industries, where controlling viscoelastic properties is critical to product performance.

### 2.2. Non-Linear Rheological Behavior of the FMP Paste

**Figure 4:**
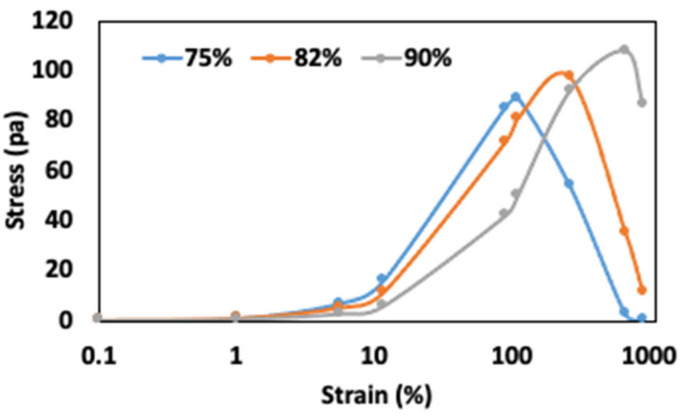
Stress vs. strain analysis of FMP paste.

The non-linear rheological behaviors of FMP paste with varying moisture contents are presented in Table 1 and Figure 4. These parameters provide valuable insights into the structural transitions of the paste under large deformations, which are critical for understanding the material’s behavior during industrial processing [36]. The stress vs. strain curves (Figure 4) reveals distinct non-linear characteristics. In the initial elastic region, all samples exhibit a linear relationship between stress and strain, with moduli of elasticity of 1.391, 0.964, and 0.493 for 75%, 82%, and 90% moisture, respectively. These values indicate that the 75% moisture sample is stiffer, requiring more stress to initiate flow compared to the more flexible 90% moisture paste.

The crossover strain, where the material transitions from solid-like (elastic) to fluid-like (viscous) behavior, further reflects these structural transitions. At 75% moisture, the crossover strain is 18%, suggesting a denser protein structure that resists deformation before becoming fluid-like. In contrast, increasing moisture content to 82% and 90% reduces the crossover strain to 17% and 16%, respectively. This trend suggests that higher moisture levels disrupt the protein entanglements and reduce structural integrity, allowing the paste to transition to a more fluid-like state under lower stress, a finding consistent with studies on soy protein and other protein-based systems where increased moisture weakens protein–protein interactions [30].

The critical strain $\left(\gamma_{c}\right)$, indicating structural breakdown onset, shows the paste’s resilience under large strains. For moisture levels of 75%, 82%, and 90%, $\gamma_{c}$ values were 4.32%, 2.63%, and 1.64%, respectively, demonstrating that higher moisture weakens the protein matrix, causing it to yield sooner. At 75% moisture, the structure, evidenced by the highest $\gamma_{c}$, supports greater deformation before breakdown, with corresponding G′ at $\gamma_{c}$ showing that FMP paste with 75% moisture has a much higher G′ (16,062 Pa) than that at 90% moisture (294 Pa), further illustrating network weakening as moisture increases.

The physical meaning of the crossover strain and $\gamma_{c}$ lies in their distinct reflection of the material’s response to large deformations. Crossover strain indicates the shift from solid-like to fluid-like behavior, while $\gamma_{c}$ marks yielding onset, where the structure starts to break down. Higher moisture content increases protein flexibility but weakens cohesive protein–protein entanglements that uphold elasticity, resulting in both lower crossover strain and $\gamma_{c}$. Similar reductions in $\gamma_{c}$ and elasticity have been observed in other protein-based materials with increasing moisture, like hydrated soy protein and gluten [37,38].

The cohesive energy density ($E_{c})$ is a measure of the energy required to disrupt the internal structure of the gel-like paste, reflecting the strength of the intermolecular forces. At 75% moisture, $E_{c}$ was 149 kJ/m^3^, indicating strong entangled protein networks exhibiting gel-like behavior. At 90% moisture, $E_{c}$ dropped sharply to 0.40 kJ/m^3^, highlighting how water disrupts protein entanglements, making the structure more prone to deformation. Such findings are relevant for food processing applications where product integrity during mixing or extrusion is critical, as seen in surimi seafood and wheat dough processing [39,40].

These non-linear results extend insights beyond those from LVR and frequency sweep analyses, which focused on small deformations. While LVR results characterized elasticity and viscosity under low strain, non-linear parameters like cohesive energy density and $\gamma_{c}$ show how the paste behaves under high strain, revealing moisture’s role in structural stability. Such insights enable better moisture control and balance between protein–protein and protein–water interactions, supporting improved FMP paste performance in industrial processes, where precise structural adjustment is essential for product quality.

### 2.3. Analysis of Normalized Lissajous–Bowditch Curves for FMP Paste

**Figure 5:**
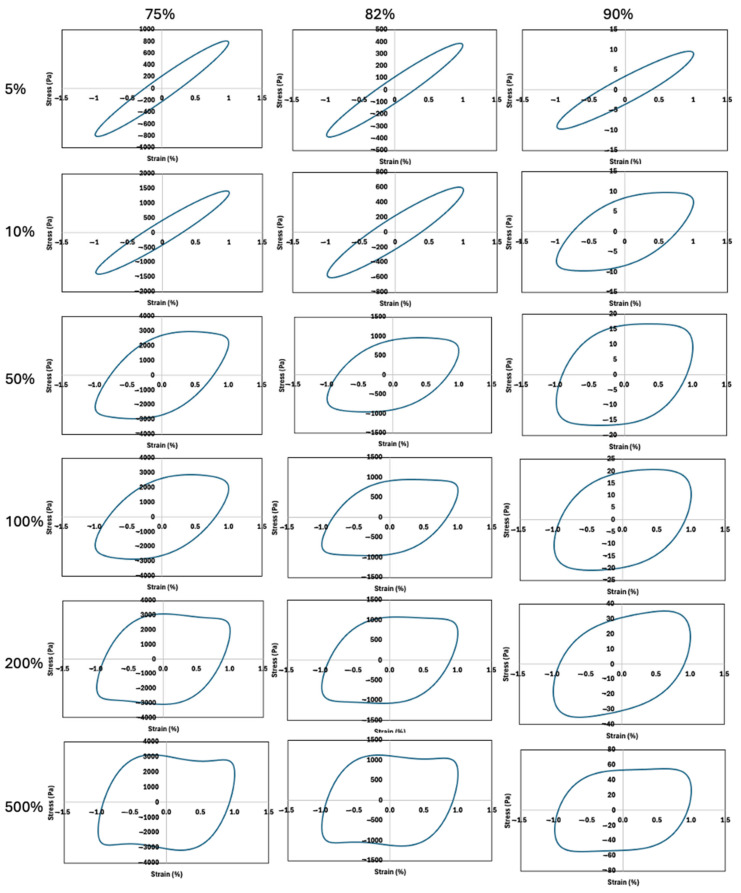
Elastic Lissajous–Bowditch plots of FMP paste at frequency of 1 rad/s and different strains of 5%, 10%, 50% 100%, 200%, and 500%. Y-axis denote shear stress (Pa), and x-axis denotes normalized strain (%).

**Figure 6:**
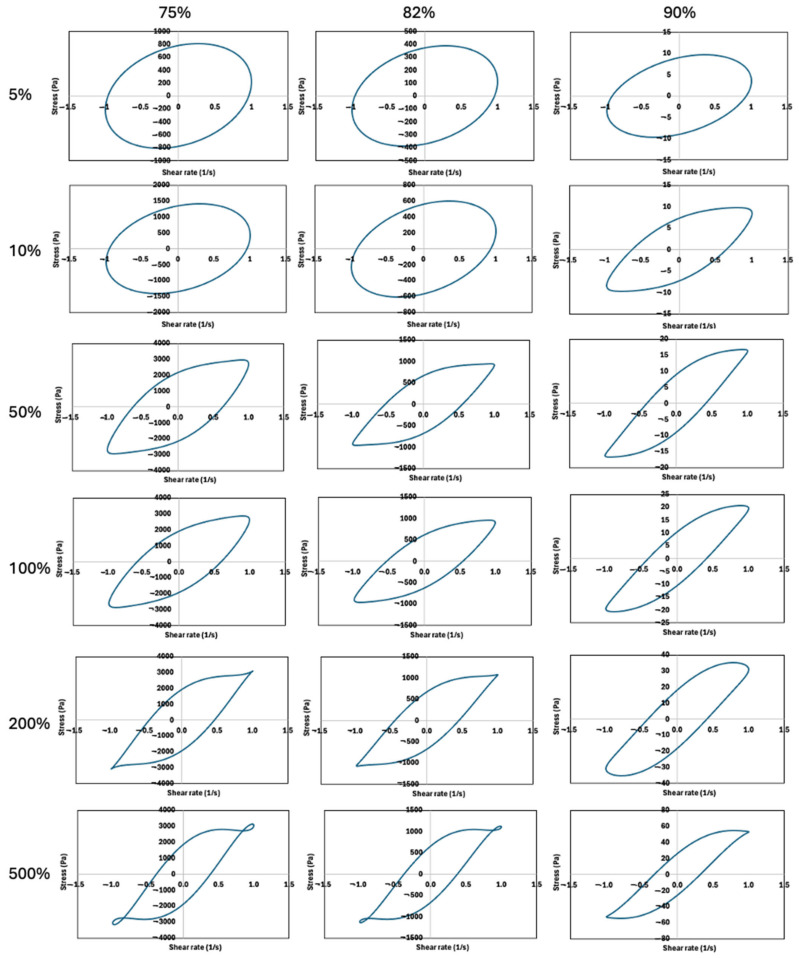
Viscous Lissajous–Bowditch plots of FMP paste at frequency of 1 rad/s and different strains of 5%, 10%, 50% 100%, 200%, and 500%. Y-axis denote shear stress (Pa), and x-axis denotes normalized shear rate (s^−1^).

LAOS tests were conducted to assess the FMP paste’s nonlinear rheological behavior under large deformations. Figure 5 and Figure 6 show the elastic and viscous Lissajous–Bowditch plots at 1 rad/s for strains of 5, 10, 50, 100, 200, and 500%. This frequency balances practical relevance with sensitivity to structural changes [13]. The Lissajous curves illustrate intracycle stress responses based on applied strain or shear rate, revealing structural transitions during large deformations [41]. At low strain (5%), the curves appear elliptical, indicating linear viscoelastic behavior dominated by elasticity within the LVR, consistent with SAOS findings [13]. With increasing strain, distortions in the curves become apparent, particularly at higher moisture levels, showing increased nonlinearity (Figure 5). These curve distortions reflect microstructural shifts, such as disrupted protein interactions and rearrangement of protein aggregates due to enhanced protein–water interactions [41,42]. Studies on soy protein and pea protein isolates similarly revealed that Lissajous curve shapes can indicate structural alterations under strain [43]. Similarly, Anvari and Joyner [41] examined fish gelatin and gum arabic emulsions and highlighted that distortions in Lissajous curves reflect the material’s capacity to undergo structural rearrangements at different strain levels.

As strain increases to 50–100% for 75% and 82% moisture content, and from 10% for 90% moisture content, curves shift to near-parallelogram shapes, showing increased viscous dissipation and a transition from weak gel-like to viscous-dominated behavior. The shift is more pronounced in samples with 90% moisture, aligning with phase angle data (Figure 2e) and suggesting significant structural changes such as breaking and reforming of protein networks with water. At 5% strain, FMP paste shows elastic straining, with yielding, flow, and structural recovery at higher strains (>10–50%). At lower moisture (75%), a dense network of protein entanglements stabilized by hydrogen bonds and hydrophobic forces is disrupted by increased strain, leading to nonlinear behavior and viscous dissipation [44]. At 90% moisture, dominant protein–water interactions weaken the network, allowing easier deformation at lower strains. Water acts as a plasticizer, loosening the structure, as reflected in more distorted Lissajous curves at lower strain (10%) [31]. Studies on other protein-based systems, such as soy and pea protein pastes, have shown similar behavior, where increased moisture content results in weakened protein networks and earlier transitions to viscous-dominated behavior [41].

The transition to S-shaped stress-strain rate loops indicates highly nonlinear viscoelastic behavior with shear-thinning at high strain rates (Figure 5). At lower strain rates (1%), all moisture levels show minimal loop distortion, while at higher strains (50–500%), nonlinear viscous contributions become prominent. The rhomboidal Lissajous curves at 10% strain in 90% moisture paste and thick curves may relate to the dispersion of myofibrillar protein aggregates in water, becoming apparent as strain rate increases. Increased strain rates result in significant shear thinning and a transition to a more viscous state due to loosened protein entanglements [41,43].

In summary, the Lissajous–Bowditch curves reveal the balance between protein–protein and protein–water interactions in FMP paste. Lower moisture levels maintain network integrity, resisting deformation, while higher moisture disrupts this network, increasing fluidity and early onset of nonlinear behavior. These insights help optimize FMP paste processing, especially in processes requiring large deformations like extrusion.

### 2.4. Analysis of Chebyshev Coefficients for FMP Paste with Varying Moisture Contents

**Figure 7:**
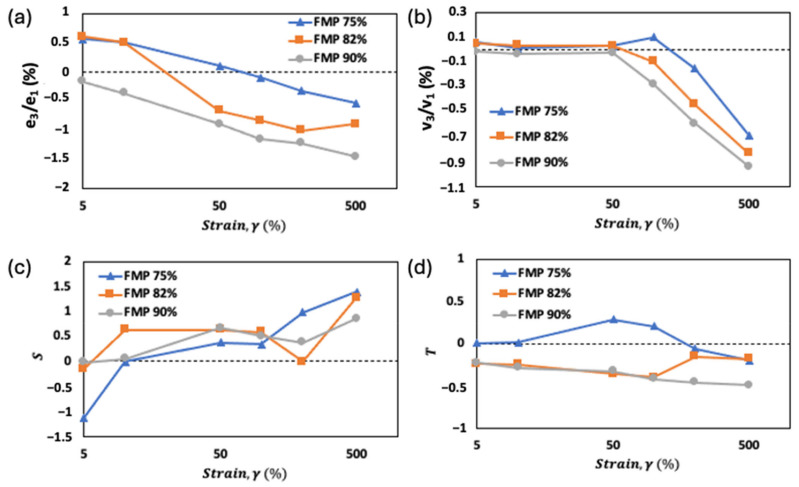
The changes in (**a**) e_3_/e_1_, (**b**) v_3_/v_1_, (**c**) S, and (**d**) T with respect to strain, γ (%), of FMP paste at different moisture contents.

The Chebyshev stress decomposition method was applied to analyze FMP paste’s viscoelastic properties through nonlinearity indicators (e_3_/e_1_, v_3_/v_1_, S, and T). Figure 7a–d show that moisture content and strain levels significantly impact these coefficients. At 75% and 82% moisture, strain stiffening (e_3_/e_1_ > 0) occurs until 50% and 10% strain, respectively, as protein chains align and stretch, forming a more rigid network due to protein–protein interactions. As strain increases further, e_3_/e_1_ becomes negative, indicating strain thinning, where the internal structure weakens and flows more easily (Figure 7a). This transition reflects the rearrangement of the internal protein network, as previously reported in protein-based systems like soy and pea protein isolates [16]. Interestingly, at 90% moisture, negative e_3_/e_1_ values appear from the start, indicating strain thinning even at low strains due to excess water acting as a plasticizer and weakening protein networks. As strain rises, e_3_/e_1_ further decreases, indicating structural relaxation [6].

Shear thickening (v_3_/v_1_ > 0) in 75% and 82% moisture samples suggests increased resistance to flow at low strains, caused by protein–protein and protein–water rearrangements (Figure 7b). At higher strains, all samples show shear thinning (negative v_3_/v_1_), facilitating easier flow under shear, advantageous for processes like extrusion and mixing due to reduced energy demands. In 90% moisture samples, consistent shear thinning highlights water’s role in lowering flow resistance, a behavior typical in high-moisture food systems. Similar observation of shear thinning behavior has been reported for surimi paste due to the breakdown of protein networks under high strain [16].

The strain-stiffening ratio (S) and shear-thickening ratio (T) values reinforce these findings (Figure 7c,d). Positive S values at higher strains in lower moisture samples confirm increased resistance to deformation under stress, while negative T values at higher moisture levels reflect a shift to shear thinning. With high moisture, water enhances molecular mobility, promoting strain thinning. Positive T values in 75% moisture samples indicate a tendency toward elastic behavior within the LVR, likely from intermolecular bonds like hydrogen bonding. At higher strains (200–500%), T values turn negative, marking a transition to strain thinning as the network structure loosens. Samples with 82% and 90% moisture show consistent strain thinning across all strain levels, with the paste becoming more fluid-like at higher deformations.

Understanding Chebyshev coefficients aids in optimizing processing conditions like screw speed, temperature, and die geometry in extrusion, mixing, and molding. High e_3_/e_1_ (strain stiffening) values support shape retention post-extrusion, crucial for structural integrity. Negative v_3_/v_1_ (shear thinning) allows easier flow, reducing energy needs in extrusion and pumping. In molding, strain-thinning behavior (negative e_3_/e_1_) requires careful control to avoid defects like incomplete filling. Analyzing these coefficients enables manufacturers to predict material behavior under processing conditions, improving product texture and consistency, and serving as a key factor in consumer acceptance.

### 2.5. Industrial Relevancce of Nonlinear Viscoelastic Properties of FMP Paste

#### 2.5.1. Extrusion Behavior and Optimization of Processing Parameters

**Figure 8:**
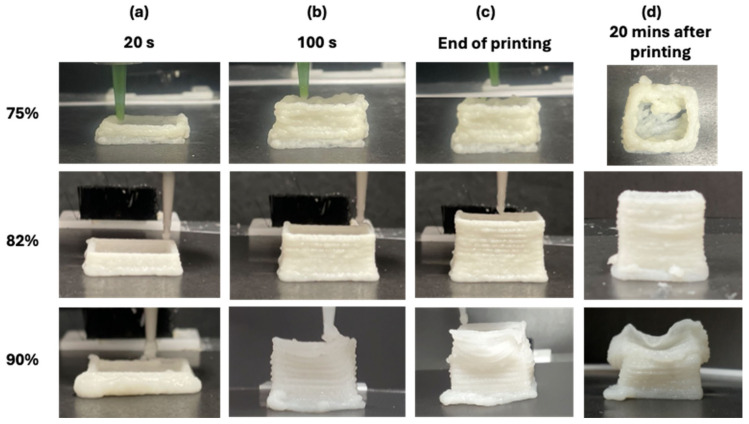
Three-dimensional-printed FMP paste at different time intervals during extrusion process. The images show the paste at (**a**) 20 s, (**b**) 100 s, (**c**) end of printing time, and (**d**) 20 min after printing.

Figure 8 and Table 2 illustrate the time-dependent variations in printability, structural stability, and die swell effect observed during the extrusion of FMP paste at different moisture levels. The 3D printing process, which imposes high strain on the material, revealed significant differences in extrusion behavior, printability, and stability across moisture contents, corresponding closely to the nonlinear viscoelastic properties identified in the rheological analysis. For the 75% moisture paste, which exhibited strong strain stiffening and elastic nonlinearity (Figure 7), the yield-stress liquid characteristics dominated its behavior under extrusion. This moisture level resulted in a highly structured material with limited flowability under prolonged printing, leading to nozzle clogging after approximately 100 s (Figure 8). The increasing stiffness at higher strains suggests that the FMP paste at 75% moisture quickly reached a point where further deformation was resisted, hindering smooth extrusion and causing interruptions. This finding is consistent with other studies that highlight the role of strain stiffening in reducing flowability, especially in high-strain environments such as 3D printing. For example, research on hydrogels has demonstrated that strain stiffening contributes to material stability but also limits its ability to flow under high strain, which could lead to issues like clogging in extrusion-based processes [44,45]. The 3D printing result of 75% moisture paste aligns with these findings, suggesting that the nonlinear viscoelastic behavior at low moisture content is crucial for predicting extrusion failures in highly structured pastes.

In contrast, 82% moisture paste demonstrated optimal printability and stability, maintaining shape integrity even 20 min post-extrusion (Figure 8). The moderate strain stiffening and shear thickening observed at this moisture level facilitated smooth extrusion while ensuring the printed structure remained stable. This balance of elastic and viscous nonlinearity supports findings in the literature, where moderate shear thickening and strain stiffening in pastes are associated with improved structural integrity during printing. Studies on dual-network hydrogels also report that these behaviors are key to achieving both good flow and stability during extrusion, preserving model fidelity [46,47]. This stability is further supported by a mid-range die swell ratio (10.19%), indicating a controlled expansion at the nozzle that contributes to model fidelity after extrusion. Thus, the nonlinear viscoelastic properties of FMP paste at 82% moisture effectively predict a favorable balance of flow and stability, making this formulation ideal for 3D printing applications requiring high strain resilience.

For the 90% moisture paste, the weak structural integrity, as reflected by low strain stiffening and near-zero thickening ratios (Figure 6), allowed for continuous extrusion, but the layers began to deform as the model reached 60% of its intended height, with significant collapse 20 min post-printing. The low die swell ratio (5.69%) further confirms the weak structural properties at this moisture level, indicating minimal resistance to deformation upon extrusion. This excessive flowability and lack of elasticity, predicted by the low strain-stiffening response, compromised the printed structure’s stability and fidelity, reinforcing that high moisture levels reduce shape retention under high strain. The nonlinear viscoelastic parameters accurately predict the failure of high moisture pastes to maintain form after extrusion, highlighting the importance of understanding material response under large deformations.

#### 2.5.2. Numerical Analysis of Deformation During 3D Printing of FMP Paste at Different Moisture Levels

**Figure 9:**
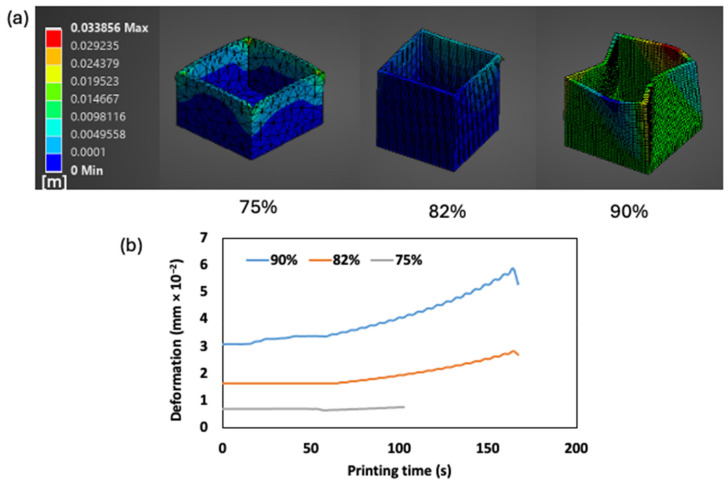
Simulated 3D printing process for FMP paste at different moisture contents. (**a**) Contour image of deformation in the completely printed model and (**b**) deformation value in the paste.

Figure 9 illustrates the deformation analysis of extruded FMP paste at different moistures during the 3D printing process. Significant differences in deformation patterns were observed, directly linked to the paste’s nonlinear viscoelastic properties and moisture content. For 75% moisture paste, the initial deformation was 0.71 × 10^−2^ mm, indicating a relatively rigid structure. The deformation remained stable until approximately 100 s into the printing process, at which point the material began exhibiting significant strain stiffening at the nozzle exit. This led to reduced flowability and eventual nozzle clogging, halting the extrusion. The paste’s yield-stress liquid characteristics contributed to this behavior, as the material quickly resisted further deformation, making continued extrusion impossible. This paste is therefore ideal for applications requiring structural stability, particularly in extrusion processes involving large nozzle sizes (> 1 mm), where the paste needs to maintain shape without excessive flow.

In the case of the 82% moisture paste, the material displayed an optimal balance of flow and stability. The initial deformation was 1.65 × 10^−2^ mm, with gradual increases (up to 2.68 × 10^−2^ mm) as the printed model height reached 58%. This increase was attributed to the growing weight of the stacked layers, but it did not result in structural failure. The moderate strain stiffening and shear thickening observed in the rheological analysis of this paste allowed for smooth extrusion, with minimal deformation and no collapse of the printed model. The paste’s ability to maintain its shape under strain was a result of the balance between its elastic and viscous properties, confirming 82% moisture content as the ideal formulation for processes requiring moderate structural stability.

In contrast, the 90% moisture paste exhibited weak strain stiffening, resulting in excessive flowability and a lack of structural stability. The deformation increased rapidly from 10 s into the printing process from 3.07 × 10^−2^ to 5.31 × 10^−2^ mm, leading to significant shape changes as more layers were added. This excessive flowability, coupled with the paste’s inability to support its own weight, caused structural collapse as the printing progressed. The lack of sufficient elasticity to maintain the model’s shape under strain was evident, leading to deformation and failure. The numerical results predicted this collapse, and the experimental results confirmed that high moisture content compromises shape retention and material stability during 3D printing. The 90% moisture paste is therefore best suited for processes where paste flow is prioritized over structural integrity.

In summary, the deformation analysis, guided by nonlinear viscoelastic properties, provides valuable insights into the printability and stability of FMP pastes. While SAOS measurements alone are limited in predicting material behavior under the high strains encountered during extrusion, the numerical analysis effectively predicted the pastes’ performance, highlighting the importance of selecting an optimal moisture content. These findings underscore the critical role of nonlinear rheology in ensuring material stability, guiding the development of reliable and consistent 3D printing processes.

## 3. Conclusions

This study highlights the critical role of nonlinear viscoelastic properties in characterizing fish myofibrillar protein (FMP) paste for industrial applications. While linear rheological tests showed differences in the storage modulus across moisture levels, nonlinear behavior provided deeper insights into material performance under strain. FMP paste with 75% and 82% moisture exhibited strain stiffening, which resisted deformation and supported structural integrity, while the 90% moisture paste demonstrated strain thinning at lower strain levels, indicating weaker structural properties. These nonlinear characteristics were crucial in understanding material behavior during 3D printing, where the 75% moisture paste experienced nozzle clogging due to rapid strain stiffening, while the 82% moisture paste showed optimal printability and stability. The Chebyshev stress decomposition method further revealed significant differences in elastic and viscous nonlinearity, emphasizing the need for advanced rheological analysis in extrusion, mixing, and molding applications. This study demonstrates that measuring nonlinear viscoelastic properties is essential for predicting material behavior under high-strain conditions, ensuring product consistency and guiding the optimization of processing parameters in manufacturing.

## 4. Materials and Methods

### 4.1. Preparation of Fish Myofibrillar Protein (FMP) Paste

A high-quality FMP from Alaska pollock (FA grade, Trident Seafood, Seattle, WA, USA) with 75% moisture, 24.1% protein, 0.9% fat, and a pH of 6.8 was obtained from Pulmuone Co., Ltd. (Gangnam-gu, Seoul, Republic of Korea). The frozen block was thawed at room temperature (25 ± 2 °C) for 4 h before being cut into 3 cm^3^ cubes. Sodium chloride (2 g/100 g of FMP) was added to promote partial solubilization of myofibrillar proteins, essential for paste network formation, as described by Park [15]. This salt enhances protein solubility and facilitates protein–protein interactions while chopping disrupts the native structure, creating a loosely bound paste network with weak gel properties. To examine the effect of moisture on rheological properties, the paste moisture content was adjusted to 82% and 90% by adding deionized water and chopping the cubes with a universal food processor (Model UMC5, Stephan Machinery Corp., Hameln, Germany) at low speed for 1 min. These moisture levels are typical in processing techniques, like co-extrusion, to improve fluidity and structural development, crucial for optimizing texture and processing efficiency in FMP products. Ice water (0 °C) was circulated around the processor to keep the sample temperature below 5 °C. The paste was stored in a plastic bag at 5 °C until analysis.

### 4.2. Small-Amplitude Oscillatory Shear (SAOS) Measurement for FMP Paste

Dynamic oscillatory measurements of FMP paste were performed using a Discovery Hybrid Rheometer HR-3 (TA Instruments, New Castle, DE, USA). Small-amplitude oscillatory shear (SAOS) was assessed with a parallel plate and Universal Peltier system (plate diameter = 40 mm), maintaining a 2 mm gap and a temperature of 5 °C to preserve the structural integrity of the Alaska pollock FMP, which can denature at higher temperatures [15]. The paste relaxed for 5 min on the Peltier plate before testing. A strain sweep analysis from 0.01% to 100% at a constant frequency of 1 Hz was conducted to determine the linear viscoelastic region (LVR), where the storage modulus (G′) and loss modulus (G″) remained relatively constant with increasing strain, indicating a linear strain–stress relationship. A dynamic frequency sweep was performed from 0.1 to 100 rad/s at a constant strain (0.1%) within the LVR, examining the elastic (G′) and viscous moduli (G″) with frequency and yielding damping factors (G″/G′) at 1, 40, and 100 rad/s. The power law model equations were used to analyze the frequency dependence of G′ and G″ [16]:

(1) $$G^{\prime }=k^{\prime }\cdot \omega^{n^{\prime }},$$

(2) $$G^{\prime \prime }=k^{\prime \prime }\cdot \omega^{n^{\prime \prime }},$$

where coefficients k′ and k″ are power law constants (Pa·s^n^), n′ and n″ are frequency exponents (dimensionless), and ω represents the angular frequency (rad/s).

### 4.3. Large-Amplitude Oscillatory Shear (LAOS) Measurement for FMP Paste

The strain sweeps for LAOS tests were conducted at a temperature of 5 °C. An oscillatory amplitude test within the range of 5% to 500% strain at a frequency of 1 rad/s was performed. The critical strain ($\gamma_{c}$) and the corresponding storage modulus $\left({G^{\prime }}_{cr}\right)$ were obtained through the experiments. The critical strain ($\gamma_{c}$) is the strain level where G′ dropped by 5% in the LVR [48]. The cohesive energy density $\left(E_{c}\right)$ which refers to the intermolecular forces holding the molecules together within the FMP paste was calculated using Equation (3) [49]:

(3) $$E_{c}=\frac{1}{2}{\gamma_{c}}^{2}{G^{\prime }}_{cr},$$

where $\gamma_{c}$ represents the critical strain, and ${G^{\prime }}_{cr}$ represents the corresponding storage modulus.

In addition, the MITlaos program (MITlaos Ver. 2.1 Beta) utilizing the nonlinear analysis introduced in Ewoldt et al. [7] was used to analyze the Lissajous curve and the discrete Chebyshev coefficient, i.e., the harmonics e_1_, e_3_, e_5_, … for the elastic domain and the harmonics v_1_, v_3_, v_5_,… for the viscous domain from the stress and strain data. The Chebyshev coefficients provided the ratios e_3_/e_1_ and v_3_/v_1_, representing the elastic component and viscous component, respectively, to characterize the rheological properties under large deformation. In the linear elastic and viscous regions, the contribution of higher harmonics is negligible, resulting in e_3_ = 0 and v_3_ = 0, respectively. Strain stiffening occurs when e_3_/e_1_ > 0, while strain thinning is characterized by e_3_/e_1_ < 0. Similarly, shear thickening corresponds to v_3_/v_1_ > 0, whereas shear thinning is indicated by v_3_/v_1_ < 0. Additionally, various nonlinear viscoelastic properties describing the characteristics of FMP paste with Chebyshev coefficients are described in Equations (4)–(9) [48,50].

(4) $${G^{\prime }}_{M}={\left.\frac{d\sigma }{d\gamma }\right|}_{\gamma =0}\approx e_{1}-{3e}_{3}+{5e}_{5}-{7e}_{7}+\ldots ,$$

(5) $${G^{\prime }}_{L}={\left.\frac{\sigma }{\gamma }\right|}_{\gamma =\pm \gamma_{0}}\approx e_{1}+e_{3}+e_{5}+e_{7}+\ldots ,$$

where e_1_, e_3_, e_5_, … represent the elastic Chebyshev coefficients previously described. Consequently, ${G^{\prime }}_{M}$ signifies the dynamic modulus at γ=0 (i.e., the maximum shear rate), while ${G^{\prime }}_{L}$ denotes the dynamic modulus at $\gamma =\pm \gamma_{0}$ (i.e., the maximum imposed strain). Similarly, the nonlinear viscous material properties at the minimum resolvable shear rate and the maximum absolute shear rate were calculated using Equations (6) and (7) [44,46]:

(6) $${\eta^{\prime }}_{M}={\left.\frac{d\sigma }{d\dot{\gamma }}\right|}_{\dot{\gamma }=0}\approx v_{1}-{3v}_{3}+{5v}_{5}-{7v}_{7}+\ldots ,$$

(7) $${\eta^{\prime }}_{L}={\left.\frac{\sigma }{\dot{\gamma }}\right|}_{\dot{\gamma }=\pm {\dot{\gamma }}_{0}}\approx v_{1}+v_{3}+v_{5}+v_{7}+\ldots ,$$

Two further ratios, i.e., the stiffening ratio (S) and the thickening ratio (T), have physical significance, and are defined by Equations (8) and (9) [44]:

(8) $$S=\frac{{G^{\prime }}_{L}-{G^{\prime }}_{M}}{{G^{\prime }}_{L}}\approx \frac{{4e}_{3}-{4e}_{5}+{8e}_{7}+\ldots }{e_{1}+e_{3}+e_{5}+e_{7}+\ldots }\;$$

(9) $$T=\frac{{\eta^{\prime }}_{L}-{\eta^{\prime }}_{M}}{{\eta^{\prime }}_{L}}\approx \frac{{4v}_{3}-{4v}_{5}+{8v}_{7}+\ldots }{v_{1}+v_{3}+v_{5}+v_{7}+\ldots }$$

These ratios are adept at capturing nonlinear behaviors of materials. Specifically, positive S values indicate strain stiffening, while negative values suggest thinning. Likewise, positive T values signify intracycle shear thickening, while negative values indicate thinning [7].

### 4.4. Three-Dimensional Printing Process of FMP Paste and Extrusion Behaviour Validation

#### 4.4.1. Three-Dimensional Printing Geometry Design and Die Swell Analysis

**Figure 10:**
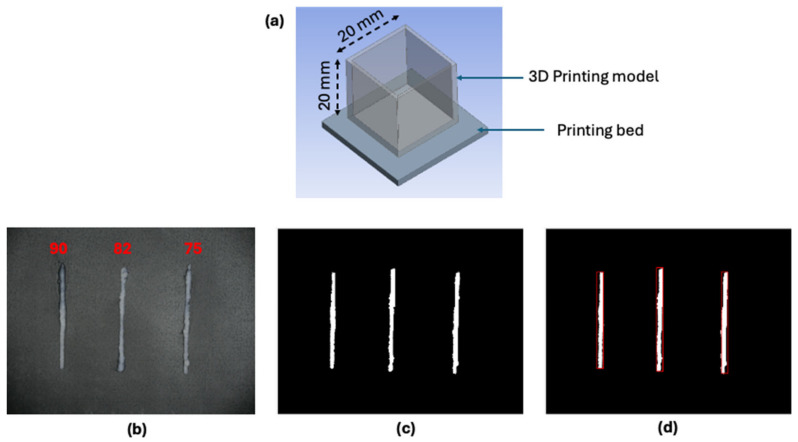
Schematic diagram of 3D printed model (**a**) and die swell analysis procedure for 3D printed FMP paste with different moisture contents: (**b**) Image of extruded paste at 50 mm length, (**c**) Binary image of extruded paste, and (**d**) Image with bounding box.

The extrusion process of FMP paste was conducted using a syringe-based fused deposition modeling (SFDM) printer (SHINNOVE-S2, Shinnove Co. Ltd., Hangzhou, Zhejiang, China), in a temperature-controlled chamber at 10 °C. Optimum printing parameters for the FMP paste (nozzle size of 1.00 mm, travel speed of 1.5 mm/s, and layer height of 1 mm) were selected based on previous studies [19]. To assess printability and structural stability, an open-box model with dimensions of 20 mm × 20 mm × 20 mm was printed (Figure 10a). For evaluating the die swell ratio at the nozzle opening, a cylindrical model (50 mm in length and 1 mm in diameter) was printed. Changes in the size of the extruded paste were recorded using a digital camera (DSLR-500D, Canon Inc., Tokyo, Japan) at a resolution of 5184 × 3888 pixels. Image processing was performed in MATLAB (MathWorks^®^ Inc., Natick, MA, USA) following these steps: (1) capturing the image, (2) extracting the image background and generating a binary image, and (3) measuring object length and width using a bounding box (Figure 10b–d). The average width of the extrudate, measured in pixels along its length, was converted to millimeters and used to calculate the die swell ratio (Equation (10)).

(10) $$Die\;swell\;ratio\;\left(\%\right)=\frac{Average\;extrudate\;width-Nozzle\;diameter}{Nozzle\;diameter}\times 100$$

#### 4.4.2. Simulation of 3D Printing Process

Following the description in [19], the 3D printing simulation was developed using a multilayer construction model designed to reflect the thermomechanical and structural properties of FMP paste. The FEM-based software additive suite 2024 R2 (Ansys, Inc., Canonsburg, PA, USA) was used to analyze the deposition process of FMP paste. The viscoelastic properties and nonlinear properties obtained from LAOS were used to prepare a material that was assigned to the 3D printing model (Figure 10a). The finite element simulation was conducted as a time-dependent model to capture the non-linear temperature field evolution accurately. Thermo-elastic structural analysis followed, estimating the deformation in the FMP paste during printing. Initially, a thermal stress model was implemented to capture environmental temperature effects on the material. This was succeeded by a coupled thermo-elastic-plastic structural analysis for the printed layers, employing transient, non-linear heat-balance equations based on Fourier–Kirchhoff heat conduction, as shown in Equation (11).

(11) $$\rho c\frac{\partial T}{\partial t}=\frac{\partial T}{\partial x}\left(k\frac{\partial T}{\partial x}\right)+\frac{\partial }{\partial y}\left(k\frac{\partial T}{\partial y}\right)+\frac{\partial }{\partial z}\left(k\frac{\partial T}{\partial z}\right)+Q_{v}$$

where ρ represents the density, c is specific heat, T is temperature, t is time, k is thermal conductivity, and $Q_{v}$ represents the volumetric heat flux. Thermal properties were measured using the KS-1 sensor with the KD2 Pro device (Decagon Devices, Inc., Pullman, WA, USA) and are presented in Table 3. For the transient thermal simulation, both the printing bed (base material, Figure 10a) and the FMP model were treated as homogeneous and isotropic materials. However, the thermophysical parameters (i.e., k, c, and ρ) varied with the sample’s moisture content.

After computing the thermal model and determining the temperature T at all nodes across different time intervals, these results were fed into the stress model. The primary equations governing the stress model are as follows:

(12) $$\delta_{ij}\mu \sigma =0\;\;\;\;$$

where $\delta_{ij}$ represents the Kronecker tensor. The total strain tensor $\varepsilon_{ij}$ consists of three components: the elastic strain $\varepsilon_{ij}^{e}$, the plastic strain $\varepsilon_{ij}^{p}$, and the thermal expansion strain $\varepsilon_{ij}^{th}$, represented as follows:

(13) $$\varepsilon_{ij}=\varepsilon_{ij}^{e}+\varepsilon_{ij}^{p}+\varepsilon_{ij}^{th}$$

The $\varepsilon_{ij}^{th}$ at temperature T was calculated by

(14) $$\varepsilon_{ij}^{th}=\alpha_{e}\Delta T=\alpha_{e}\left(T-T_{ref}\right)$$

where $T_{ref}$ represents the reference temperature (i.e., 10 °C) and $\alpha_{e}$ represents the linear coefficient relating to the rate at which strain changes with a unit temperature. The constitutive model of the total stress $\sigma_{ij}$ and elastic strain $\varepsilon_{hk}^{e}$ is:

(15) $$\sigma_{ij}=C_{ijhk}\varepsilon_{hk}^{e}$$

where $C_{ijhk}$ denotes the fourth-order elasticity tensor as a function of the elastic modulus E and Poisson’s ratio v, obtained from [19].

### 4.5. Statistical Analysis

All treatments were conducted on at least three freshly prepared FMP pastes. The mean values and standard deviations of each treatment were calculated after 5 repetitions of the experiment for each treatment. The SPSS 19.0 statistical analysis program was used to analyze the data, and the results were presented as mean values ± standard deviations.

## Figures and Tables

**Table 1:** Crossover strain (%), critical strain $\left(\gamma_{c}\right)$, the corresponding storage modulus $\left({G^{\prime }}_{cr}\right)$ at critical strain, and the cohesive energy density of FMP paste at different moisture contents.

Moisture Content of FMP (%)	Crossover Strain (%)	Critical Strain, $\gamma_{c}$ (%)	G′ at Critical Strain, ${G^{\prime }}_{cr}$ (Pa)	Cohesive Energy Density, $E_{c}$ (kJ/m^3^)
75	18 ± 1.12 ^a^	4 ± 0.67 ^a^	16,062 ± 22 ^a^	149 ± 0.002 ^a^
82	17 ± 0.88 ^a^	2. ± 0.42 ^b^	5730 ± 10 ^b^	19 ± 0.001 ^b^
90	16 ± 0.22 ^b^	1 ± 0.51 ^c^	294 ± 3 ^c^	0.40 ± 0.001 ^c^

Values within the same column with different superscripts (a, b, and c) differ significantly (*p* < 0.05).

**Table 2:** Die swell analysis of extruded FMP paste from 1 mm nozzle size.

	Moisture Content of FMP Paste (%)		
	75	82	90
Average thickness across extrudate (mm)	1.133 ± 0.03 ^c^	1.102 ± 0.02 ^b^	1.057 ± 0.03 ^a^
Die swell ratio (%)	13.32	10.19	5.69

Values within the same row with different superscripts (a, b, and c) differ significantly (*p* < 0.05).

**Table 3:** Thermal properties and density of FMP paste at 10 °C.

	Moisture Content of FMP Paste (%)		
	75	82	90
Thermal conductivity ${(W\cdot m}^{-1}\cdot K^{-1})$	0.587	0.534	0.530
Thermal resistivity $\left({m\cdot K\cdot W}^{-1}\right)$	252.92	249.85	248.33
Specific heat ${(J\cdot K}^{-1}\cdot {Kg}^{-1})$	3427	3682	3791
Density ${(g\cdot cm}^{-3})$	1.09	1.03	1.01

## Data Availability

Data are available on request from the corresponding author.
